# Ultrasound- and Microbubble-Assisted Gemcitabine Delivery to Pancreatic Cancer Cells

**DOI:** 10.3390/pharmaceutics12020141

**Published:** 2020-02-07

**Authors:** Tormod Bjånes, Spiros Kotopoulis, Elisa Thodesen Murvold, Tina Kamčeva, Bjørn Tore Gjertsen, Odd Helge Gilja, Jan Schjøtt, Bettina Riedel, Emmet McCormack

**Affiliations:** 1 Department of Medical Biochemistry and Pharmacology, Haukeland University Hospital, Bergen 5021, Norway; tina.kamceva@helse-bergen.no (T.K.); jan.didrik.schjott@helse-bergen.no (J.S.); bettina.riedel@helse-bergen.no (B.R.); 2 Department of Clinical Science, Faculty of Medicine, University of Bergen, Bergen 5021, Norway; bjorn.gjertsen@uib.no; 3 Phoenix Solutions AS, Ullernchausseen 64, 0379 Oslo, Norway; spiros.kotopoulis@uib.no; 4 National Centre for Ultrasound in Gastroenterology, Haukeland University Hospital, Bergen 5021, Norway; odd.helge.gilja@helse-bergen.no; 5 Department of Clinical Medicine, University of Bergen, Bergen 5021, Norway; 6 KinN Therapeutics AS, Bergen 5021, Norway; Elisa.Thodesen@uib.no; 7 Department of Internal Medicine, Hematology Section, Haukeland University Hospital, Bergen 5021, Norway; 8 Centre for Cancer Biomarkers CCBIO, Department of Clinical Science, University of Bergen, Bergen 5021, Norway

**Keywords:** gemcitabine, sonoporation, pancreatic cancer, PDAC, hENT, nucleoside transporters, in vitro

## Abstract

Pancreatic ductal adenocarcinoma (PDAC) is a major cause of cancer death worldwide. Poor drug delivery to tumours is thought to limit chemotherapeutic treatment efficacy. Sonoporation combines ultrasound (US) and microbubbles to increase the permeability of cell membranes. We assessed gemcitabine uptake combined with sonoporation in vitro in three PDAC cell lines (BxPC-3, MIA PaCa-2 and PANC-1). Cells were cultured in hypoxic bioreactors, while gemcitabine incubation ± sonoporation was conducted in cells with operational or inhibited nucleoside membrane transporters. Intracellular active metabolite (dFdCTP), extracellular gemcitabine, and inactive metabolite (dFdU) concentrations were measured with liquid chromatography tandem mass spectrometry. Sonoporation with increasing US intensities resulted in decreasing extracellular gemcitabine concentrations in all three cell lines with inhibited membrane transporters. In cells with inhibited membrane transporters, without sonoporation, dFdCTP concentrations were reduced down to 10% of baseline. Sonoporation partially restored gemcitabine uptake in these cells, as indicated by a moderate increase in dFdCTP concentrations (up to 37% of baseline) in MIA PaCa-2 and PANC-1. In BxPC-3, gemcitabine was effectively inactivated to dFdU, which might represent a protective mechanism against dFdCTP accumulation in these cells. Intracellular dFdCTP concentrations did not change significantly following sonoporation in any of the cell lines with operational membrane transporters, indicating that the gemcitabine activation pathway may have been saturated with the drug. Sonoporation allowed a moderate increase in gemcitabine transmembrane uptake in all three cell lines, but pre-existing nucleoside transporters were the major determinants of gemcitabine uptake and retention.

## 1. Introduction

Pancreatic ductal adenocarcinoma (PDAC) is one of the leading causes of cancer death worldwide [1,2]. Late stage diagnosis precludes surgical excision in the majority of patients [3], and poor drug delivery into the tumour tissue limits chemotherapeutic efficacy in patients with advanced disease [4,5,6].

Gemcitabine monotherapy is one of the three main chemotherapeutic drug regimens used in the palliative setting of PDAC patients worldwide [7]. Following cellular uptake, primarily via the equilibrative nucleoside transporter 1 (hENT1), gemcitabine is either inactivated by cytidine deaminase (CDA) to 2′,2′-difluoro-2′-deoxyuridine (dFdU) and effluxed or phosphorylated through a series of nucleoside kinases to active metabolites. Deoxycytidine kinase (dCK), which catalyses the initial phosphorylation of gemcitabine to gemcitabine monophosphate (dFdCMP), is a rate limiting enzyme in the activation pathway [8]. The main active metabolite, gemcitabine triphosphate (dFdCTP), exerts its activity by inhibiting DNA replication [8]. Expression levels of hENT1 [9,10], CDA [11], and dCK [10] in tumour tissue have been associated with gemcitabine efficacy.

Paproski and co-workers demonstrated that in vitro inhibition of hENT1 by dilazep reduced average gemcitabine uptake 24-fold and sensitivity 13-fold in both PDAC and non-PDAC cell lines. Restoration of nucleoside membrane transport by transfection with an active nucleoside influx pump re-established gemcitabine uptake and sensitivity [12], suggesting that hENT1 was a major mediator of gemcitabine transport across cell membranes. Conversely, CP4126 a lipidic derivative of gemcitabine, which elicits its effect independent of hENT1, failed to demonstrate benefit versus gemcitabine in a Phase III clinical trial (trial number NCT00913198, clinicaltrials.gov). Macrophages [13,14], fibroblasts [2], and bacteria [15,16] in the tumour microenvironment have also been suggested to modulate gemcitabine efficacy in PDAC. Moreover, limited drug delivery to PDAC tumours has been postulated to confer treatment failure [9].

The combination of microbubbles and ultrasound (US) has been proposed to facilitate the formation of transient pores in biological membranes through a process commonly referred to as sonoporation [17], resultantly permitting increased tissue drug delivery and cellular uptake of drugs. In a phase 1 clinical trial, PDAC patients (*n* = 10) were treated with gemcitabine followed by repeated intravenous boluses of SonoVue^®^ microbubbles and US focused at their primary tumours. Sonoporation treated patients experienced tumour shrinkage, tolerated an increased number of treatment cycles, and survived longer than a historical control group, of comparable performance status, treated with gemcitabine alone [18]. Similar results were achieved in a preclinical trial in mice with orthotopic PDAC xenografts [19]. It was postulated that the observed effects might partly be explained by increased gemcitabine delivery to PDAC tumour cells. This hypothesis was based on prior in vitro experiments in which cell-impermeable fluorescent drug surrogates had been shown to enter cells exposed to sonoporation [20,21,22].

Mariglia and co-workers [23], however, found no increase in intracellular uptake and retention of a radiolabelled nucleoside analogue similar to gemcitabine, following in vitro sonoporation of a suspension of the PDAC cell line BxPC-3. The authors proposed that direct cellular effects of sonoporation, rather than an increase of gemcitabine delivery, could potentially explain an additive cytotoxicity which was observed with Definity^®^ microbubbles and increasing US intensities, employing a frequency of 0.5 MHz and mechanical indices (MI) of 0.31–0.50–0.75, ISPPA 1.61–4.32–9.36 W/cm^2^ and ISPTA 0.052–0.14–0.30 W/cm^2^ [23]. The ultrasound settings were within the clinical diagnostic limits. However, their study was limited to a single cell line. Differences between cell lines regarding activities in hENT1 and enzymes involved in drug-metabolism, such as CDA, have not been evaluated in previous sonoporation studies of gemcitabine [18,19,23]. We, therefore, assessed in vitro uptake and metabolism of gemcitabine in three adherent PDAC cell lines, with and without inhibition of hENTs and CDA, following incubation with therapeutically relevant drug concentrations, commercially available microbubbles and diagnostic US intensities.

We hypothesised that the effect of sonoporation on cellular gemcitabine uptake could depend on the activities of hENTs or gemcitabine metabolizing enzymes.

## 2. Materials and Methods

### 2.1. Chemicals and Reagents

Chemicals and reagents were purchased from Merck KGaA (Darmstadt, Germany) unless otherwise stated, and were of analytical grade. Culture flasks and cryotubes were purchased from VWR (Oslo, Norway), centrifuge tubes from Sarstedt (Oslo, Norway) and Petaka^®^ G3 LOT (Celartia, Columbus, OH, USA) hypoxic cell culture bioreactors (hereafter entitled “Petakas”) from Tebu-Bio (Roskilde, Denmark). Horse serum and sodium pyruvate were obtained from Thermo Fisher Scientific (Oslo, Norway) and tetrahydrouridine (THU) from AH diagnostics (Oslo, Norway). Reagents and equipment used for liquid chromatography tandem mass spectrometric methods (LC-MS/MS) are described elsewhere [24,25].

### 2.2. Cell Culture

The PDAC cell lines, BxPC-3, MIA PaCa-2 and PANC-1, were kindly provided by Prof. Anders Molven (University of Bergen, Bergen, Norway). Cell lines had been authenticated by DNA-fingerprinting [26] and was used within 15 passages after thawing. BxPC-3 were cultured in Roswell Park Memorial Institute 1640 medium (RPMI) and MIA PaCa-2 and PANC-1 in Dulbecco’s Modified Eagles Medium (DMEM) in a humidified atmosphere with 5% CO_2_ at 37 °C. Media were complemented with 4 mM sodium pyruvate, 2 mM L-glutamine and 10% fetal bovine serum (FBS). Horse serum (2.5%) was added to the medium used for MIA PaCa-2. No antibiotics were used. Mycoplasma-tests performed on a regular basis were negative.

**Figure 1:**
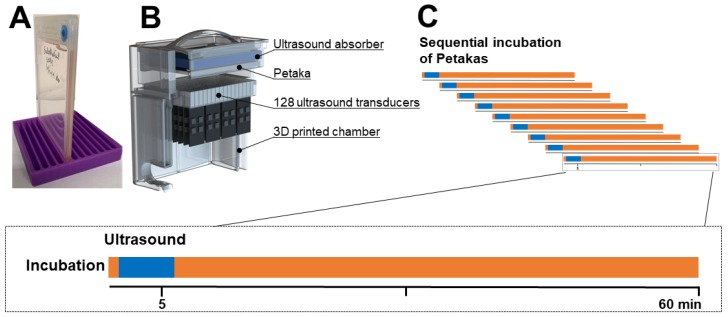
Experimental procedure and timeline. In each batch of experiments, Petaka^®^ G3 LOT hypoxic bioreactors (**A**) were sequentially incubated to avoid concurrency conflicts (**C**). 1 mL culture medium with the appropriate gemcitabine concentrations and Sonazoid^®^ microbubbles were injected through the injection port. Immediately following injection, the Petakas were transferred to the custom-made ultrasound treatment chamber (**B**), sonicated for five minutes (indicated by blue in the timelines) and returned to the incubator. Culture media and trypsinised cells were aspirated through the injection port after incubation with gemcitabine for 60 min (indicated by orange in the timelines).

Two or three days before experiments with gemcitabine, cells were harvested using 0.05% trypsin-EDTA, counted and reseeded into Petakas (Figure 1A) at a density of 2.0–4.0 × 10^6^ cells per 25 mL medium. Petakas were kept in a horizontal position for 24 h to ensure even cell distribution over the surface, and then flipped to a vertical position with the air vent at the top, until the day of the experiments. At the day of experiments, cell confluency averaged 70–80%. *A priori* evaluation of cell growth had been performed for each cell line at four different seeding densities, and surface area coverage was quantified using MIPAR^™^ image analysis software [27] (Supplemental Figure S1).

### 2.3. Gemcitabine Incubation and Sonoporation

Three series of sonoporation experiments were performed in all three cell lines: (1) 60 min incubation with 10 µM gemcitabine, (2) 20 min pre-incubation with 100 µM dilazep followed by 60 min incubation with 10 µM gemcitabine and (3) 60 min co-incubation with 10 µM gemcitabine and 200 µM tetrahydrouridine (THU), an inhibitor of cytidine deaminase (CDA).

In all experiments, we used one microbubble concentration and selected US intensities based on a priori optimisation, with the cell-impermeable dye calcein as “drug surrogate” (Supplemental Figures S2 and S3). Sonazoid^®^ was prepared using the venting needle method. A total of 2 mL of saline (B.Braun AG, Melsungen, Germany) was slowly added to the vented vial and gently agitated for 30 s. Eighty µL Sonazoid^®^ stock solution with 1.20 × 10^9^ microbubbles per mL was added to 1 mL of the prepared gemcitabine solution, and injected into the Petakas. Air pockets were removed and the entire Petaka was exposed to US immediately thereafter. The Petakas (Figure 1A) were placed in the water bath of a custom-made US treatment system, with the cell monolayer on the upper surface to maximise cell-microbubble contact. The US treatment system (Figure 1B) was based on a previous design [28] and consisted of 128, 9 × 6 mm PZ26 elements firing upwards in groups of 16 elements at a time as a plane-wave into the Petaka. The distance between the ultrasound transducer and absorber was 27 ± 1 mm. The US transducers were driven by a custom Open Ultrasound system (Lecoeur Electronique, Chuelles, France). The acoustic field had been calibrated in the fully assembled US chamber in three axes using a 200-µm needle hydrophone (Precision acoustics Ltd., Dorset, United Kingdom). The Petaka was placed at the acoustic focus. Ultrasound was applied for a total of 5 min at a frequency of 2.0 MHz. Two acoustic intensity levels were applied: **Medium** (MI 0.2, 80 cycles, duty cycle (DC) 1.8%, *I*_SPPA_ 3 W/cm^2^ and *I*_SPTA_ 50 mW/cm^2^) and **High** (MI 0.378, 160 cycles, DC 3.6%, *I*_SPPA_ 10 W/cm^2^ and *I*_SPTA_ 358 mW/cm^2^), in addition to **Control** (no US). An ultrasound frequency of 2 MHz was chosen as this is commonly used in non-linear contrast imaging for Sonazoid microbubbles. In addition, this frequency is below the resonance frequency of Sonazoid, and would ensure the bubbles to resonate in phase with the ultrasound, maximising volumetric oscillations [29]. The medium ultrasound intensity was chosen to mimic pulse lengths of previous experiments [19]. The high ultrasound intensity was chosen as this was the maximum output the open ultrasound system could produce in the given configuration. The temperature in the water bath was monitored using an analogue alcohol thermometer. After treatment, Petakas were returned to the incubator until completion of 60 min gemcitabine incubation time (Figure 1C). At the end of experiments, 1 mL of medium was collected, transferred to cryotubes and kept at −80 °C until quantification of extracellular gemcitabine and dFdU. The adherent cells were rinsed with phosphate-buffered saline (PBS), trypsinised and re-suspended in cold culture medium. Cells were counted and centrifuged at 1250 RPM for five minutes. Supernatants were discarded and cell pellets were either diluted and reseeded in 24-well plates for postexposure cell growth assays, or dissolved in cold 60% methanol, transferred to cryovials, vortexed for 20 s, snap-frozen in liquid nitrogen and stored at −80 °C until quantification of intracellular dFdCTP.

### 2.4. Quantification of Gemcitabine and Its Metabolites

Quantification of gemcitabine and its metabolites was performed using an Agilent 1200 series HPLC-system (Agilent Technologies, Waldbronn, Germany) for chromatographic separation and an Agilent 6410 triple-quad mass spectrometer for mass detection. Concentrations of gemcitabine and dFdU in culture media samples were measured according to our previously published method [24], with optimised lower limits of quantitation (LLOQ) of 0.1 µM for both analytes. Gemcitabine triphosphate (dFdCTP) was quantified in cell lysates with a slightly modified version of our previously published method [25]. Modification consisted of reduced analysis time to approximately 30 min and with the mass spectrometer operating in positive ionisation mode, since we only quantified dFdCTP and not the endogenous nucleosides that eluted later. dCTP was used as internal standard due to its similar structure and retention time with dFdCTP. Concentrations above the LLOQ of 0.05 µM were normalised to the cell count in each sample and expressed as pmol per 10^6^ cells (abbreviated to pmol/10^6^ throughout the manuscript).

### 2.5. Cell Growth after Incubation with Gemcitabine ± Sonoporation

Cell viability following exposure to (1) 60 min 10 µM gemcitabine alone, (2) sonoporation (High) alone, (3) 60 min 10 µM gemcitabine combined with sonoporation (High), and (4) Control (drug-free media, i.e., untreated cells), was assessed by monitoring cell growth for up to ten days. BxPC-3 suspensions were diluted to 2500 cells/mL, MIA PaCa-2 and PANC-1 to 1000 cells/mL, and reseeded in triplicate into 24-well plates. Five daily snapshots from each well were captured using a Zeiss Vert.A1 microscope, Axiocam 105 colour camera and the Zeiss ZEN Pro 2012 blue edition software. Images (*n* = 3600 in total) were analysed using MIPAR™ image analysis software Version 3.0 [27]. Cell growth over time was expressed as percentage surface area coverage.

### 2.6. Statistical Analyses

Quantitative data were processed using Microsoft Office Excel (2016) and GraphPad Prism 8 (San Diego, CA, USA). All experiments were performed with *n* = 3/4 at each experimental condition in all three cell lines, with a total of approximately 200 Petakas used. Variations of measurements were expressed as mean ± standard deviations (SD). Two-sided independent student’s *t*-tests were used to compare means between experiments performed at two different ultrasound acoustic intensities (Medium or High) or no ultrasound (Control), within each cell line. Correction for multiple testing was not performed in this explorative in vitro study where the number of groups did not exceed 3 in any cell line or any experiment. A one-tailed Pearson’s correlation was used to describe linear relationships between US intensities and gemcitabine and—metabolite concentrations within each cell line. One-tailed was based on the assumption that increasing US intensities would have a one-directional effect on gemcitabine and—metabolite concentrations. Pearson’s was based on the assumption that the measures of US intensities, MI and *I*_SPTA_, represented continuous variables to be examined for a linear relationship to gemcitabine and metabolite concentrations. A *p*-value less than 0.05 was considered significant.

## 3. Results

### 3.1. Sonoporation and Cellular Gemcitabine Uptake

**Figure 2:**
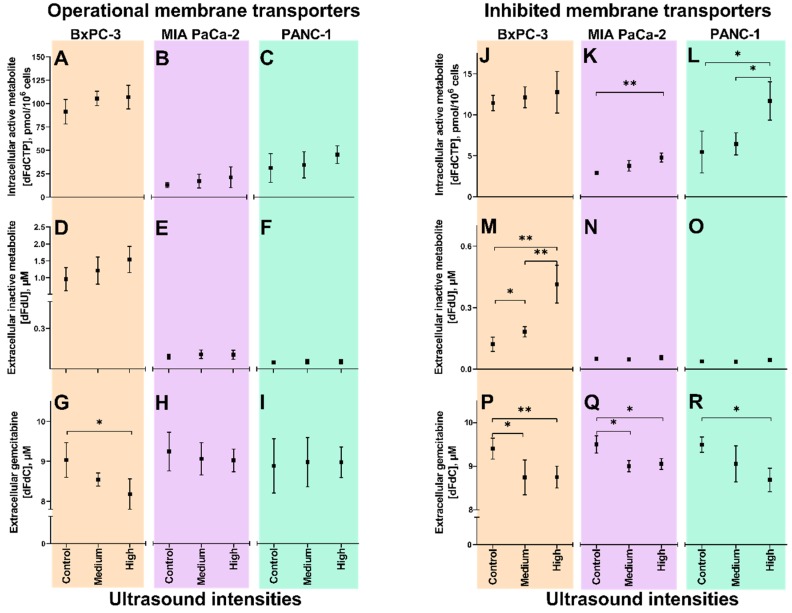
Gemcitabine uptake and metabolism following sonoporation of pancreatic ductal adenocarcinoma (PDAC) cell lines with operational and inhibited membrane transporters. Extracellular concentrations of gemcitabine (dFdC, panels **G**, **H**, **I** and **P**, **Q**, **R**), extracellular inactive metabolite (dFdU, panels **D**, **E**, **F** and **M**, **N**, **O**) and intracellular active metabolite (dFdCTP, panels **A**, **B**, **C** and **J**, **K**, **L**) in BxPC-3 (orange), MIA PaCa-2 (purple) and PANC-1 (green). Results displayed as mean±SD (*n* = 3–4). * *p* < 0.05, ** *p* < 0.01 (Unpaired students *t*-tests). Notice the different scales on the *Y*-axes of extracellular dFdU and intracellular dFdCTP concentrations in experiments with operational vs. inhibited membrane transporters. **“Operational membrane transporters”** (panels **A**–**I**): 60 min incubation with 10 µM gemcitabine, 3.84 × 10^6^ ppmL Sonazoid^®^ microbubbles and 5 min ultrasound (US) at two acoustic intensities (Medium^1^, High^2^) and no US (Control), in Petakas. **“Inhibited membrane transporters”** (panels **J**–**R**): 20 min pre-incubation with 100 µM dilazep followed by 60 min incubation with 10 µM gemcitabine, 3.84 × 10^6^ ppmL Sonazoid^®^ microbubbles and 5 min US at two acoustic intensities (Medium^1^, High^2^) and no US (Control), in Petakas. ^1^ Medium US intensity: 2.0 MHz, MI 0.2, 80 cycles, DC 1.8%, *I*_SPPA_ 3 W/cm^2^ and *I*_SPTA_ 50 mW/cm^2^; ^2^ High US intensity: 2.0 MHz, MI 0.378, 160 cycles, DC 3.6%, *I*_SPPA_ 10 W/cm^2^ and *I*_SPTA_ 358 mW/cm^2^.

**Figure 3:**
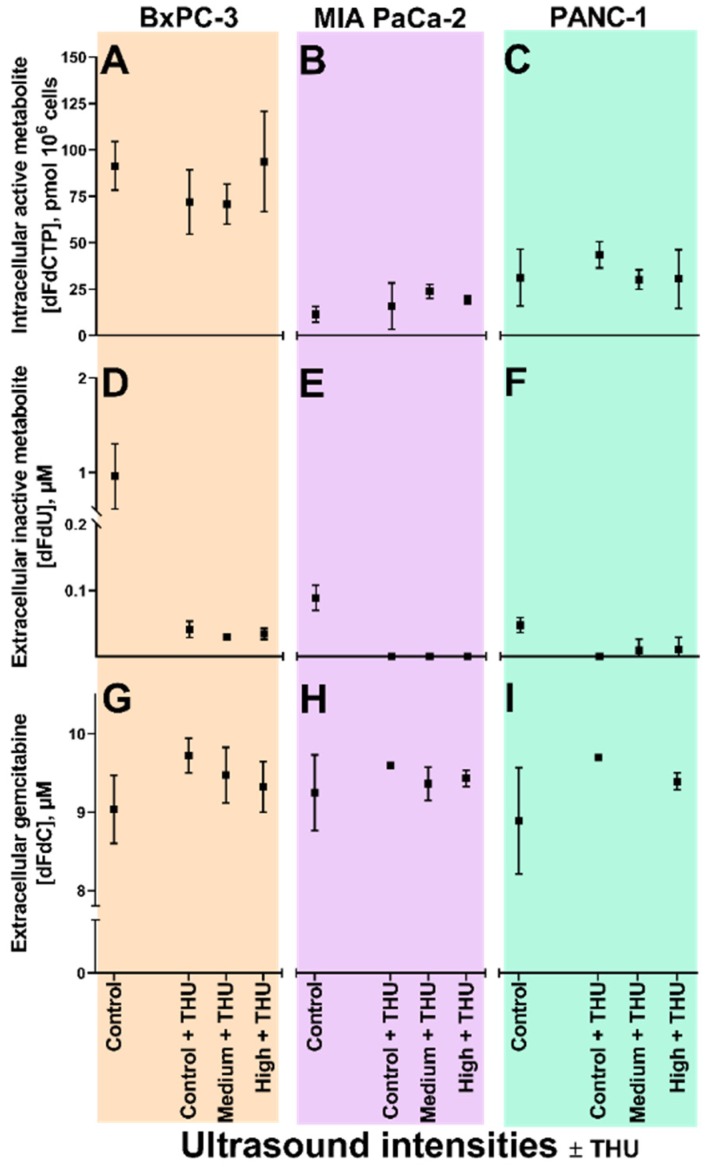
Gemcitabine uptake and metabolism following sonoporation of PDAC cell lines with inhibited cytidine deaminase. Extracellular gemcitabine (dFdC, panels **G**, **H**, **I**), extracellular inactive gemcitabine metabolite (dFdU, panels **D**, **E**, **F**), and intracellular active gemcitabine metabolite (dFdCTP, panels **A**, **B**, **C**), in BxPC-3 (orange), MIA PaCa-2 (purple) and PANC-1 (green) cell lines following 60 min co-incubation with 10 µM gemcitabine +200 µM tetrahydrouridine (THU), 3.84 × 10^6^ ppmL Sonazoid^®^ microbubbles and 5 min ultrasound (US at two acoustic intensities (Medium^1^, High^2^) and no US (Control) in Petakas. 60 min incubation with 10 µM gemcitabine without US included as control (leftmost data point in all panels). Results displayed as mean ± SD (*n* = 3–4). No significant differences between means of Control + THU vs. Medium + THU vs. High + THU (Unpaired students *t*-tests). ^1^ Medium US intensity: 2.0 MHz, MI 0.2, 80 cycles, DC 1.8%, *I*_SPPA_ 3 W/cm^2^ and *I*_SPTA_ 50 mW/cm^2^; ^2^ High US intensity: 2.0 MHz, MI 0.378, 160 cycles, DC 3.6%, *I*_SPPA_ 10 W/cm^2^ and *I*_SPTA_ 358 mW/cm^2^.

All three cell lines were incubated with gemcitabine and Sonazoid^®^ microbubbles, and treated with US at medium and high intensities, and without US (control). The contribution of membrane transporter activities in the cellular uptake of gemcitabine combined with sonoporation was elucidated by gemcitabine incubation ± the hENT-inhibitor dilazep. Data from cells with operational membrane transporters are displayed in Figure 2A–I, and with inhibited membrane transporters in Figure 2J–R. The impact of intracellular cytidine deaminase activities on the outcome of gemcitabine uptake ± sonoporation was also assessed, and results are displayed in Figure 3.

### 3.2. Sonoporation of Cells with Operational Membrane Transporters

In BxPC-3, after 60 min incubation with 10 µM gemcitabine and with application of the highest US intensity, mean extracellular gemcitabine concentrations were reduced from 9.0 ± 0.4 µM (Control) to 8.2 ± 0.4 µM (*p* = 0.025) (Figure 2G). Extracellular dFdU (Figure 2D) and intracellular dFdCTP (Figure 2A) showed a trend towards higher concentrations, but the observed changes were not statistically significant (*p* = 0.07 and *p* = 0.14 for dFdU and dFdCTP, respectively). A significant correlation was however observed between gemcitabine concentrations and MI (*p* = 0.017, *r*^2^ = 0.997), and dFdU concentrations and MI (*p* = 0.035, *r*^2^ = 0.988) in BxPC-3.

In MIA PaCa-2 and PANC-1, no significant changes were observed in gemcitabine or -metabolite concentrations following sonoporation. A significant correlation was, however, observed between dFdCTP concentrations and MI (*p* = 0.005, *r*^2^ = 1.000) in MIA PaCa-2 (Figure 2B). In PANC-1, no correlations between concentrations of gemcitabine or -metabolites and US intensities were observed.

### 3.3. Inhibition of Membrane Transporters

Cells were incubated for 60 min with 10 µM gemcitabine, with or without 20 min pre-incubation with 100 µM dilazep [12]. Without US (Control), in BxPC-3, extracellular dFdU concentrations were reduced from 1.0 µM without dilazep (Figure 2D) to 0.1 µM with dilazep (Figure 2M). In MIA PaCa-2 and PANC-1, dFdU concentrations were already low at baseline, and no further reductions could be quantified. In all three cell lines, intracellular dFdCTP concentrations were significantly reduced by dilazep: from 91.3 (Figure 2A) to 11.4 pmol/10^6^ (Figure 2J) in BxPC-3, from 12.9 (Figure 2B) to 2.9 pmol/10^6^ (Figure 2K) in MIA PaCa-2 and from 31.2 (Figure 2C) to 5.5 pmol/10^6^ (Figure 2L) in PANC-1.

### 3.4. Sonoporation of Cells with Inhibited Membrane Transporters

#### 3.4.1. Extracellular Gemcitabine

**Figure 4:**
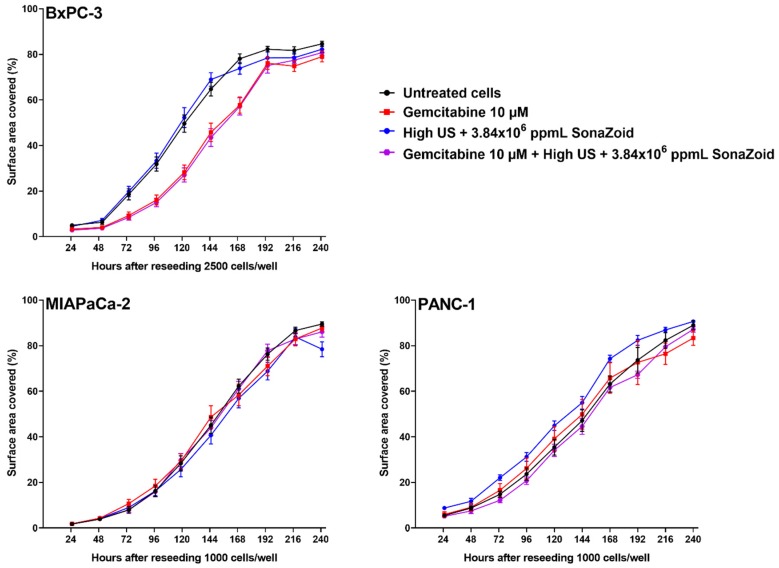
Growth of cell lines up to ten days after exposure to gemcitabine, sonoporation, or both, compared to untreated cells. Cell lines were incubated with 10 µM gemcitabine and 3.84 × 10^6^ ppmL Sonazoid^®^ microbubbles for 60 min, 5 min ultrasound (High^1^), both, or no treatment (control). Cell growth was monitored after re-seeding^2^ the cells in triplicate in 24 well-plates, and five daily images were captured from each well, using a Zeiss Vert.A1 microscope, Axiocam 105 colour camera and the Zeiss ZEN Pro 2012 blue edition software. Images were analysed with MIPAR™ image analysis software, and cell growth over time was expressed as surface area coverage. Results displayed as mean ± SD (*n* = 6). Examples of original images and analysis of surface area coverage 72 h after reseeding are given in Supplemental Figure S4. ^1^ High US intensity: 2.0 MHz, MI 0.378, 160 cycles, DC 3.6%, *I*_SPPA_ 10 W/cm^2^ and *I*_SPTA_ 358 mW/cm^2^; ^2^ Seeding densities: BxPC-3 2500 cells/well, MIA PaCa-2 and PANC-1 1000 cells/well.

In all three cell lines, following preincubation with dilazep, small but significant decreases in extracellular gemcitabine concentrations from approximately 9.5 µM without US to below 9.0 µM with increasing US intensity were noted (Figure 2P–R). Inverse correlations between gemcitabine concentrations and MI were observed for MIA PaCa-2 (*p* = 0.006, *r*^2^ = 1.00) (Figure 4Q) and PANC-1 (*p* = 0.006, *r*^2^ = 1.00) (Figure 4R).

#### 3.4.2. Extracellular dFdU

In BxPC-3, extracellular dFdU concentrations increased from 0.1 ± 0.04 (Control) to 0.2 ± 0.03 µM at medium US intensity (*p* = 0.03) and further to 0.4 ± 0.09 µM at high intensity (*p* = 0.001) (Figure 2M). This trend showed a correlation with the *I*_SPTA_ (*p* = 0.02, *r*^2^ = 0.995). No changes in dFdU concentrations were observed in MIA PaCa-2 (Figure 2N) or PANC-1 (Figure 2O).

#### 3.4.3. Intracellular dFdCTP

Intracellular dFdCTP concentrations increased from 2.9 ± 0.2 (Control) to 4.8 ± 0.6 pmol/10^6^ at high US intensity (*p* = 0.005) in MIA PaCa-2 (Figure 2K) and from 5.5 ± 2.6 to 11.7 ± 2.4 pmol/10^6^ (*p* = 0.036) in PANC-1 (Figure 2L). In BxPC-3, a small, statistically insignificant (*p* = 0.367) increase from 11.4 ± 0.9 (Control) to 12.8 ± 2.5 pmol/10^6^ at high US intensity was noted (Figure 2J). However, linear correlations between dFdCTP concentrations and MI were observed in BxPC-3 and MIA PaCa-2 (*p* = 0.0006, *r*^2^ = 1.00 and *p* = 0.0249, *r*^2^ = 0.994, respectively), whereas in PANC-1 a correlation was seen between dFdCTP and *I*_SPTA_ (*p* = 0.0063, *r*^2^ = 1.00).

### 3.5. Sonoporation of Cells with Inhibited Cytidine Deaminase

Sixty minutes co-incubation with 10 µM gemcitabine and 200 µM THU resulted in dFdU concentrations below LLOQ (<0.1 µM) in all three cell lines (Figure 3D–F). Without US, no significant differences in extracellular gemcitabine (Figure 3G–I) or intracellular dFdCTP (Figure 3A–C) concentrations were seen with or without THU added. There was also no significant change in dFdCTP concentrations in any of the three cell lines co-incubated with gemcitabine and THU when US intensity was increased.

### 3.6. Cell Growth after Exposure to Gemcitabine and/or Sonoporation

Growth of the cell lines was followed for ten days after exposure to 10 µM gemcitabine, sonoporation (High), or both, and compared to untreated cells (Figure 4). In MIA PaCa-2 and PANC-1, no differences between treatment groups was delineated. BxPC-3 cells that had been incubated with gemcitabine, either alone or combined with sonoporation, showed an initially slower growth compared to untreated cells (Figure 4, upper left). When fitting the growth curves of BxPC-3 to a 4-point logistic curve, the groups treated with gemcitabine had significantly different points of inflection compared to untreated cells and those with sonoporation alone (*p* < 0.0001), but the growth rate (Hill slope) was the same for all groups (*p* = 0.942).

## 4. Discussion

To our knowledge, this is to date the most comprehensive in vitro study of gemcitabine cellular uptake combined with sonoporation, using diagnostic intensity US and microbubbles. The majority of in vitro sonoporation studies have used cell-impermeable fluorescent drug surrogates as indicators of membrane permeation. However, the extent of cellular chemotherapeutic drug uptake is rarely reported. This study fills into this knowledge-gap by quantitating extra- and intracellular concentrations of gemcitabine and metabolites. Moreover, our data demonstrate that gemcitabine uptake and metabolite accumulation following sonoporation depend on the activities of membrane transporters and metabolizing enzymes within the cells.

### 4.1. Extracellular Gemcitabine Concentrations

In BxPC-3 with operational membrane transporters (Figure 2G), and in all three cell lines when membrane transporters had been inhibited prior to gemcitabine incubation (Figure 2P–R), extracellular gemcitabine concentrations decreased with increasing US intensities. Decreasing gemcitabine concentrations indicated that sonoporation enhanced transmembrane gemcitabine transport, since cellular uptake was the only possible route of drug removal from the media in our experimental system.

### 4.2. Significance of Membrane Transporters

Our results indicated that sonoporation contributed only to a small proportion of cellular gemcitabine uptake compared to pre-existing nucleoside membrane transporters (hENT). When hENTs had been inhibited, dFdCTP concentrations were reduced to approximately 10–20% (Figure 2J–L) of those in cells with operational membrane transporters (Figure 2A–C). This substantiated the idea of hENTs being the main determinants of gemcitabine uptake and ultimately of cellular accumulation of dFdCTP, which is also in accordance with previous studies [12,30]. Sonoporation partially restored the supply of gemcitabine in transport- inhibited MIA PaCa-2 and PANC-1, reflected by significant increases in dFdCTP concentrations from Control to High ultrasound intensities (Figure 2K,L). In both cell lines, these concentrations were 37.5% of those achieved in cells with operational membrane transporters incubated with gemcitabine, but without sonoporation (Figure 2B,C). In BxPC-3, however, where CDA is highly expressed [31], the increased gemcitabine uptake resulted in an increase in the inactive metabolite (dFdU) (Figure 2M) and no significant change in dFdCTP concentrations was noted (Figure 2J). The Pearson’s correlations of MI and/or *I*_SPTA_ and dFdCTP concentrations in all three cell lines suggest that higher US intensities may be warranted [22] in order to increase gemcitabine delivery in cells with deficient membrane transporters.

### 4.3. Gemcitabine Concentrations and Enzyme Saturation

When CDA was inhibited (Figure 3), conversion of gemcitabine to dFdU was abolished in all three cell lines. In BxPC-3, in which a priori CDA-activity was extensive, we had speculated whether the inhibition would allow more gemcitabine to be metabolised to dFdCTP. However, no increase in dFdCTP was noted, neither in BxPC-3, nor in the other cell lines. This may indicate that the activation pathway was already saturated with gemcitabine, which is in line with dCK being a rate-limiting enzyme in this pathway [32,33,34]. Indeed, the experiments with CDA inhibition were only performed in cells with operational membrane transporters which would allow continuous gemcitabine uptake from the medium, and therefore with limited additional effect of sonoporation. Whether a more pronounced effect of sonoporation could have been unmasked if cells were incubated with gemcitabine concentrations lower than 10 µM, further below a potential saturation of dCK [32,33,34], remains to be investigated.

### 4.4. Duration of Incubation

Previous findings suggest that the sonoporation effect has a duration of up to and exceeding one hour [20,35,36], supports our choice of 60 min drug incubation time in our experiments. Also, shorter incubation times could have been relevant in order to establish a dynamic range and thus detect more subtle changes in sonoporation-induced cellular gemcitabine uptake. It is likely that a major proportion of gemcitabine transport across a permeabilised membrane would occur within seconds-to-minutes after initiation of drug incubation [37,38]. Theoretically, if transport of gemcitabine through sonoporation-induced pores during this short timescale was dominant, and hENT-mediated transport reached diffusion equilibrium later, early differences between cells ± sonoporation would remain undetected. Drug-incubation and use of Petakas is expensive, laborious and time-consuming. Resultantly, seconds-to-minutes experiments were experimentally unfeasible. Nevertheless, the use of such hypoxic bioreactors was necessary to mimic the hypoxic nature of the PDAC tumours. Also, since our final outcome measure was intracellular dFdCTP concentrations, a combined marker of cellular drug uptake and subsequent phosphorylation, 60 min gemcitabine incubation time was considered to be rational [38].

### 4.5. Cellular Responses to Sonoporation and Gemcitabine

Growth curves over a 10-day period after exposure (Figure 4) indicated that BxPC-3 was more sensitive to gemcitabine than the other two cell lines. This agrees with the higher concentrations of dFdCTP in this cell line, compared to the other two cell lines (Figure 2A–C). Sonoporation, however, had no effect on cell growth over a 10-day period in any of the cell lines. We had shown in cells with operational membrane transporters, that sonoporation did not increase intracellular dFdCTP concentrations. This is in line with our observation that cell growth was not inhibited under these experimental conditions. However, cellular effects following a single 60-min incubation with gemcitabine and sonoporation with diagnostic intensity US might be more subtle than what can be observed with a growth assay. Mariglia and co-workers [23] used the MTT-assay 48 h after sonoporation, and observed decreasing cell viability with increasing US intensities in suspended BxPC-3 cells. Definity^®^ microbubbles used by Mariglia and co-workers are smaller and stiffer than the Sonazoid^®^ microbubbles used in our study, but they also used higher MIs that are known to induce inertial cavitation. Furthermore, Definity^®^ has a much more neutral zeta potential compared to Sonazoid™ (−4.2 for Definity^®^ vs. −82mV for Sonazoid™) [39,40] which may result in a very different interaction with the cells. Similar to SonoVue^®^, Definity^®^ is a very unstable microbubble, in which the concentration and size distribution changes over time i.e., bubble dissolution rate varies depending on size, changing the concentration and bulk resonance frequency. These multiple differences in physicochemical characteristics make it difficult to directly determine a primary reason for the difference in response. In addition, the Definity^®^ microbubbles were driven at 0.5 MHz which is more than 20 times lower than their fundamental resonance frequency [41]. This suggests that the microbubble behaviour may be significantly different between our study and the study by Mariglia and co-workers, making it difficult to directly compare them. Furthermore, temperatures were between 25–30 °C in our experiments, depending on room temperature, which indicated that no harmful heating of the cells would occur.

To mimic useable clinical imaging frame rates (20–30 frames per second), the ultrasound treatment system was set to not receive and store any reflected ultrasound signal; hence we were unable to confirm if the ultrasound-induced inertial or stable cavitation. Based on literature values, at the medium ultrasound setting (MI = 0.2) we expect stable cavitation, whilst at the at the high ultrasound setting (MI = 0.378) we expect stable-inertial cavitation [42]. It is important to note that the cavitation threshold is heavily influenced by the physiological conditions, hence this needs to be confirmed via acoustic spectroscopy or high-speed imaging.

### 4.6. Implications, Strengths and Limitations

The majority of in vitro research on US and microbubble assisted drug delivery has been performed using fluorescence labelled dyes that have no routes of spontaneous cellular entry [20,21,22]. As such, they are ideal model drugs for mechanistic studies and for optimisation of sonoporation settings. Methods for semi-quantitative measurements of these compounds, such as flow cytometry, are readily available. However, cell impermeable compounds are unlikely to represent all relevant properties of therapeutically active drugs. Cellular drug uptake occurs via transmembrane transport proteins or via diffusion through the lipid bilayer and might be counterbalanced by passive or active efflux [43,44,45,46,47]. As we have demonstrated, sonoporation-induced cellular uptake of gemcitabine was lower than the uptake mediated via nucleoside membrane transporters. This would not have been recognised by using cell impermeable model drugs alone. Their widespread application and the use of percentage “positive” cells in most studies, rather than quantitation of cellular drug concentrations, might even have contributed to exaggerated conclusions in terms of quantitative significance of sonoporation-induced drug uptake.

Studying sonoporation and gemcitabine-uptake in PDAC cells cultured in hypoxic Petakas is of particular interest. It has been demonstrated that cellular responses to sonoporation depend on the condition of the cells [48,49], which may be relevant for PDAC tumours when nutrient and oxygen supplies are limited [50]. Zhang and co-workers [51] showed that hypoxia-induced perturbations in endogenous nucleotide pools, and they suggested that the efficacy and toxicity of nucleoside analogues such as gemcitabine would be modified accordingly. Moreover, US assisted drug delivery is not only a product of membrane pore formations; it has also been shown to interfere with the intracellular cytoskeleton [21], that might theoretically regulate membrane transport proteins [52]. Most authors studying sonoporation, including Mariglia and co-workers [23], have reported results from cancer cell lines in suspension. In Petakas, the PDAC cells were treated while adherent. This may represent a more relevant condition compared to suspended cells in which the cytoskeleton might already have been rearranged prior to sonoporation [53]. Moreover, the impact of ultrasound treatment time on gemcitabine uptake needs to be evaluated in future studies.

Experiments performed on plastic surfaces that do not mimic either mechanical or acoustic characteristics of tissue may increase acoustic aberration [54]. Furthermore, the static in vitro environment does not mimic the blood flow seen in vivo. A dynamic blood flow would drastically reduce the contact between microbubbles and cells [55] and also affect how the cells grow [20]. The protein concentration in cell culture media are also low compared to blood, meaning the microbubbles may have an increased stability as the proteins reduce the hydrophobicity of the lipids [56]. In vivo, microbubbles would not be directly in contact with the PDAC cells but initially with endothelial cells [55], hence the effect on the PDAC cells may be lower than in vitro. In addition, the pancreatic cancer microenvironment includes other cell types such as fibroblasts [2], macrophages [13,14], typically displaying a desmoplastic reaction. Cells and surrounding tissue may be differentially affected both by gemcitabine and sonoporation, and as a result, the treatment outcome could theoretically also be influenced through effects on these cells/tissues.

## 5. Conclusions and Future Perspectives

Sonoporation with diagnostic intensity US and Sonazoid microbubbles allowed a moderate increase in gemcitabine transmembrane uptake in all three cell lines, but pre-existing nucleoside transporters were the major determinants of gemcitabine uptake and retention. Cell growth after a single treatment with sonoporation combined with gemcitabine was well preserved, which may reflect a general treatment resistance in these cell lines. Moreover, the data underscore that specific PDAC cell lines may respond differently to sonoporation due to different intracellular gemcitabine metabolism.

Future studies should include cells of multiple different origins, since a single response on a given cell line or drug does not represent a universally valid effect. Furthermore, sonoporation should be evaluated by using therapeutic drugs in more complex PDAC models that include multiple cell types, connective tissue components, and perfusion.

## Supplementary Materials

The following are available online at https://www.mdpi.com/1999-4923/12/2/141/s1, Figure S1: Growth curves of untreated BxPC-3, MIA PaCa-2 and PANC-1, Figure S2: Calcein uptake following in vitro sonoporation of BxPC-3, MIA PaCa-2 and PANC-1, Figure S3. Gating strategy for quantitation of % calcein positive cells, Figure S4: Images of BxPC-3, MIA PaCa-2 and PANC-1 reseeded after sonoporation (Raw data for Figure 4).
