# Supplementary material for: Ultrasound- and Microbubble-Assisted Gemcitabine Delivery to Pancreatic Cancer Cells
Source: Pharmaceutics. 2020 Feb 7;12(2):141. doi: 10.3390/pharmaceutics12020141 (PMC7076495; doi:10.3390/pharmaceutics12020141)
Supplement: Supplementary file 1 [file pharmaceutics-12-00141-s001.pdf]

pharmaceutics

MDPI

Supplementary Materials: Ultrasound- and Microbubble-Assisted Gemcitabine Delivery to Pancreatic Cancer Cells

Tormod Bjånes, Spiros Kotopoulis, Elisa Thodesen Murvold, Tina Kamčeva, Bjørn Tore Gjertsen, Odd Helge Gilja, Jan Schjøtt, Bettina Riedel and Emmet McCormack

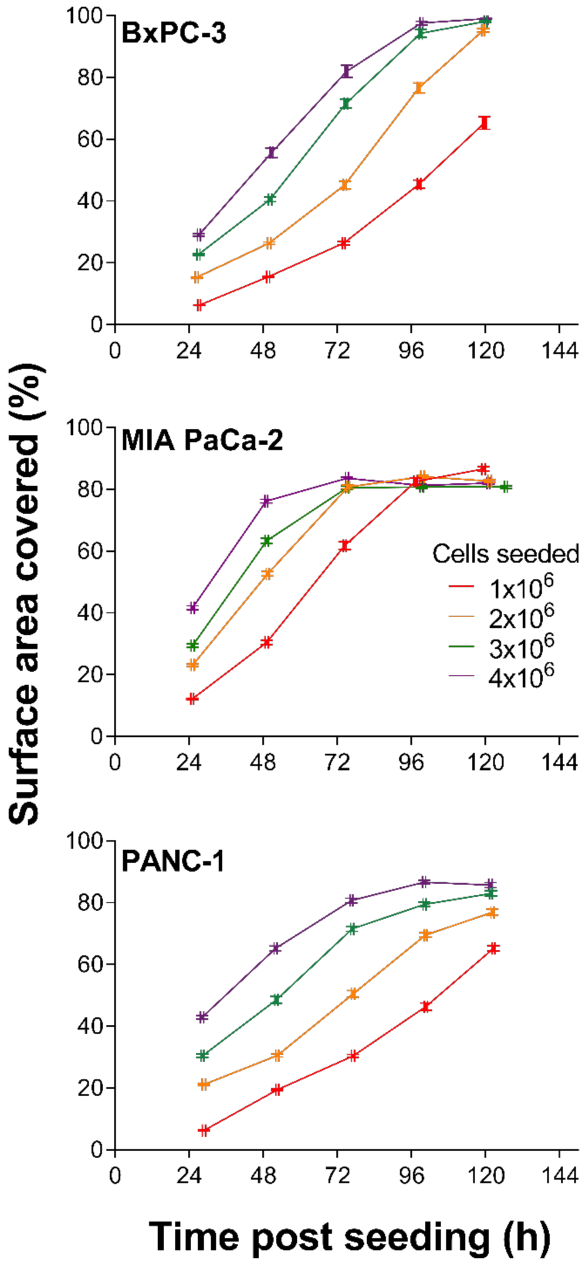

Figure S1. Growth curves of untreated BxPC-3, MIA PaCa-2 and PANC-1.
Cells were seeded in Petakas at densities from 1.0 – 4.0 x 10^6 cells in 25 mL medium. 60 daily images were captured across the surface of the Petakas using Zeiss Vert.A1 microscope, Axiocam 105 colour camera and the Zeiss ZEN Pro 2012 blue edition software. Images were analysed with MIPAR™ image analysis software and growth was expressed as surface area coverage (%) over time.

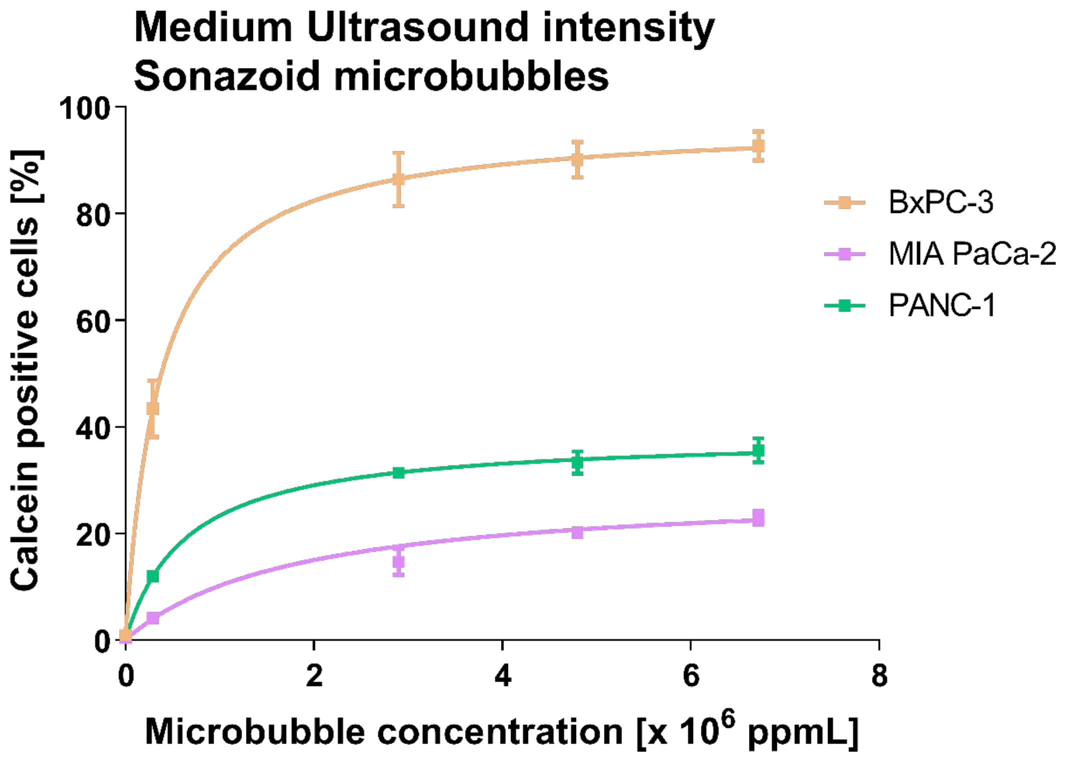

Figure S2. Calcein uptake following *in vitro* sonoporation of BxPC-3, MIA PaCa-2 and PANC-1. Example of sonoporation-induced uptake of cell-impermeable calcein at Medium[1] ultrasound intensity and increasing concentrations of Sonazoid® microbubbles in all three cell lines, assessed by flow-cytometry (mean±SD, n=3). Increase in % calcein positive cells was considered to be an indicator of increasing efficacy of cell membrane permeation. The gating strategy is shown in Supplemental Figure S3.

[1]2.0 MHz, MI 0.2, 80 cycles, DC 1.8 %, I_{SPPA} 3 W/cm^2 and I_{SPTA} 50 mW/cm^2.

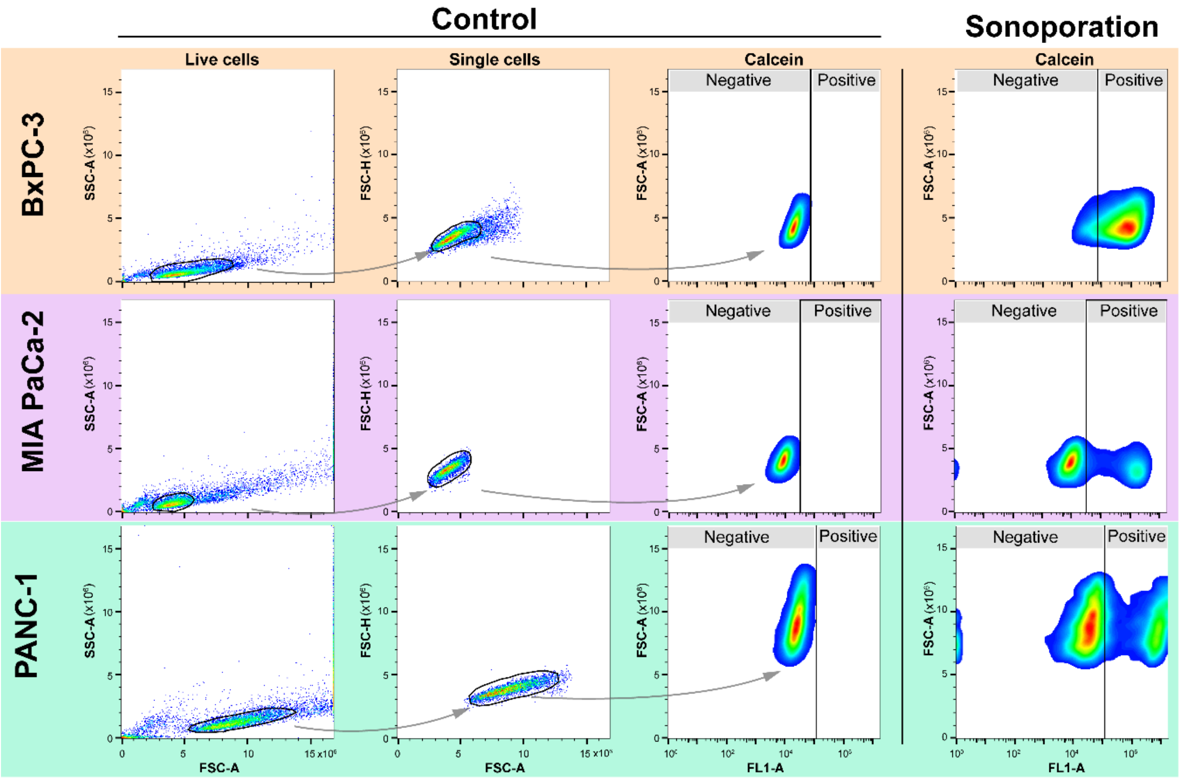

Figure S3. Gating strategy for quantitation of % calcein positive cells.
Gating strategy of flow cytometry data, illustrated by examples from all three cell lines, BxPC-3, MIA PaCa-2 and PANC-1. After identification of the live and single cell populations, the threshold for calcein positive cells was determined in the "Control" sample and subsequently used for the treated samples. The percentage of calcein positive cells was assessed after 5 minutes sonoporation at medium[1] ultrasound intensity with Sonazoid™ and 60 minutes incubation with 5 µM calcein in Petakas ("Sonoporation").

[1]2.0 MHz, MI 0.2, 80 cycles, DC 1.8 %, I_{SPPA} 3 W/cm² and I_{SPTA} 50 mW/cm².

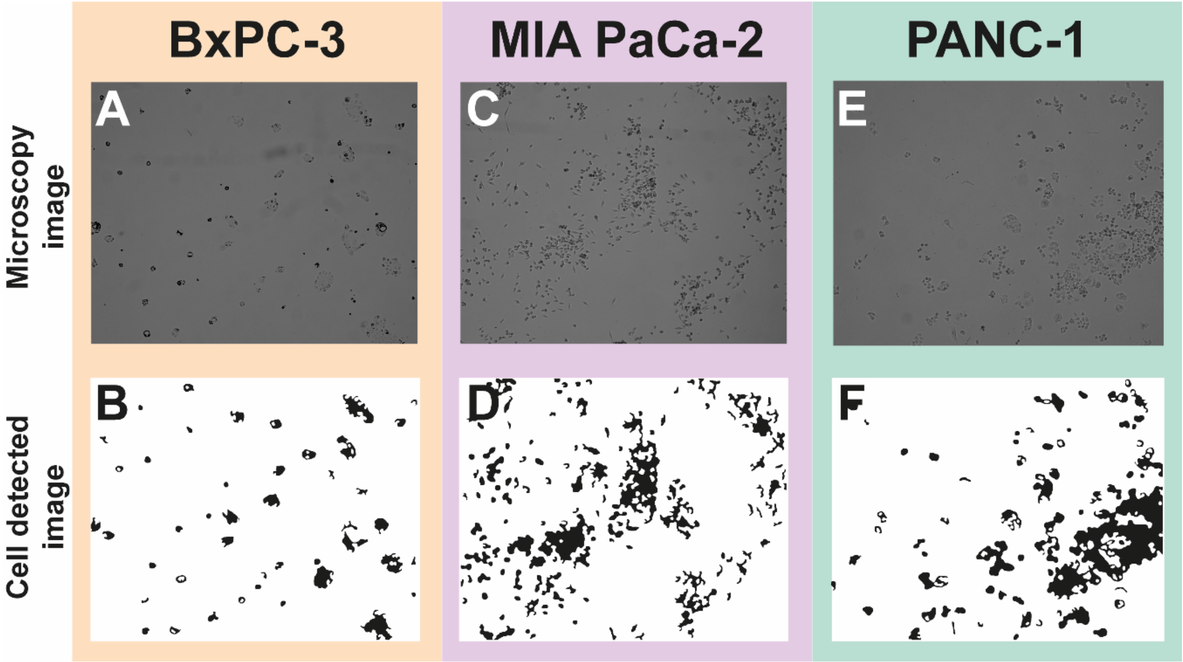

Figure S4. Example of images and analysis results for cell growth of BxPC-3 (A&B), MIA PaCa-2 (C&D) and PANC-1 (E&F) reseeded[1] after sonoporation at high[2] ultrasound intensity.

The top row (A,C, & E) show raw microscopy images for each of the cell lines, captured 72 hours after reseeding using Zeiss Vert.A1 microscope, Axiocam 105 colour camera and the Zeiss ZEN Pro 2012 blue edition software. The bottom row (B, D, & F) show the binary output images from MIPAR™ image analysis software after image recognition. The black pixels denote "detected cells". The percentage of black pixels in each image denoted the percentage of surface area covered in Figure 4.

[1] Seeding densities: BxPC-3 2500 cells/well, MIA PaCa-2 and PANC-1 1000 cells/well

[2] High US intensity: 2.0 MHz, MI 0.378, 160 cycles, DC 3.6 %, I_{SPPA} 10 W/cm^2 and I_{SPTA} 358 mW/cm^2
